# The Compound Chinese Medicine “Kang Fu Ling” Protects against High Power Microwave-Induced Myocardial Injury

**DOI:** 10.1371/journal.pone.0101532

**Published:** 2014-07-03

**Authors:** Xueyan Zhang, Yabing Gao, Ji Dong, Shuiming Wang, Binwei Yao, Jing Zhang, Shaohua Hu, Xinping Xu, Hongyan Zuo, Lifeng Wang, Hongmei Zhou, Li Zhao, Ruiyun Peng

**Affiliations:** 1 Department of Experimental Pathology, Beijing Institute of Radiation Medicine, Beijing, People’s Republic of China; 2 Department of Pharmacy, 66400 People’s Liberation Army Hospital, Beijing, People’s Republic of China; University of Catania, Italy

## Abstract

**Background:**

The prevention and treatment of Microwave-caused cardiovascular injury remains elusive. This study investigated the cardiovascular protective effects of compound Chinese medicine “Kang Fu Ling” (KFL) against high power microwave (HPM)-induced myocardial injury and the role of the mitochondrial permeability transition pore (mPTP) opening in KFL protection.

**Methods:**

Male Wistar rats (100) were divided into 5 equal groups: no treatment, radiation only, or radiation followed by treatment with KFL at 0.75, 1.5, or 3 g/kg/day. Electrocardiography was used to Electrophysiological examination. Histological and ultrastructural changes in heart tissue and isolated mitochondria were observed by light microscope and electron microscopy. mPTP opening and mitochondrial membrane potential were detected by confocal laser scanning microscopy and fluorescence analysis. Connexin-43 (Cx-43) and endothelial nitric oxide synthase (eNOS) were detected by immunohistochemistry. The expression of voltage-dependent anion channel (VDAC) was detected by western blotting.

**Results:**

At 7 days after radiation, rats without KFL treatment showed a significantly lower heart rate (P<0.01) than untreated controls and a J point shift. Myocyte swelling and rearrangement were evident. Mitochondria exhibited rupture, and decreased fluorescence intensity, suggesting opening of mPTP and a consequent reduction in mitochondrial membrane potential. After treatment with 1.5 g/kg/day KFL for 7 d, the heart rate increased significantly (P<0.01), and the J point shift was reduced flavorfully (P<0.05) compared to untreated, irradiated rats; myocytes and mitochondria were of normal morphology. The fluorescence intensities of dye-treated mitochondria were also increased, suggesting inhibition of mPTP opening and preservation of the mitochondrial membrane potential. The microwave-induced decrease of Cx-43 and VDAC protein expression was significantly reversed.

**Conclusion:**

Microwave radiation can cause electrophysiological, histological and ultrastructural changes in the heart. KFL at 1.5 g/kg/day had the greatest protective effect on these cardiovascular events. mPTP plays an important role in the protective effects of KFL against microwave-radiation-induced myocardial injury.

## Introduction

With the advent of wireless technology, there has been a massive increase of electromagnetic radiation exposure to human beings from microwaves to radio waves and other invisible radiation. It has been known that certain intensities of microwave radiation, especially high power microwave (HPM), could damage multiple organs, including heart [1]–[10]. However, the prevention and treatment of HPM-caused cardiovascular injury remains elusive. In recent years, the favorable preclinical results have been obtained with some natural compounds from traditional Chinese medicine [8], [11]. Towards that end, there is an urgent need to gain insights into the molecular basis of their effects and develop novel therapies for microwave-radiation–related myocardial injury.

In this study, we focused on “Kang Fu Ling” (KFL), a compound isolated from Chinese medicine astragalus, red peony, salvia, ophelia, and wolfberry. By using a rat microwave radiation model, we showed that KFL has myocardial protective effects by electrophysiology testing and histological analysis. Further investigation of molecular mechanisms by which pathogenesis is prevented by KFL revealed prevention of mitochondrial dysfunction may be the key event involved in KFL’s myocardial protection effects.

The increased risk of damage to the mitochondria is associated with the massive opening of mitochondrial permeability transition pore (mPTP) under pathological conditions of mitochondrial calcium overload [12]–[14]. We have found previously that KFL significantly decreased calcium level and activities of serum myocardial enzymes, such as creatine kinase-MB (CK-MB), lactate dehydrogenase (LDH), and aspartate aminotransferase (AST), etc, after microwave radiation [15]. Thus, we hypothesized now that the inhibition of mPTP opening by KFL may be responsible for its myocardial protection effects from HPM-induced myocardial injury. Indeed, in this study we found that changes of several mPTP associated protein expression after microwave radiation could be reverted by KFL treatment. This study sheds new light on the role of mPTP in mitochondrial dysfunction and molecular basis of KFL protection.

## Materials and Methods

### Ethics Statement

All animal procedures were performed in accordance to the guidelines of the laboratory animal centre of Beijing Institute of Radiation Medicine, following approval of the Animal Welfare and Ethics Committee of Beijing Institute of Radiation Medicine. All surgery was performed under sodium pentobarbital anesthesia and all efforts were made to minimize suffering.

### Animals

A total of 100 male Wistar adult rats weighing 160±20 g were randomly divided into 5 groups: normal controls (C), radiation (R), KFL low dose: 0.75 g/kg/day (L), KFL medium dose: 1.5 g/kg/day (M), and KFL high dose: 3 g/kg/day (H) (n = 20 per group). Animals obtained from the Laboratory Animal Center (Beijing, China) were housed five per cage and maintained at 22±2°C with a 12 hour light-dark cycle (lights on at 7 a.m.) and access to food and water *ad libitum* within the observation period.

### Microwave radiation

**Figure 1:**
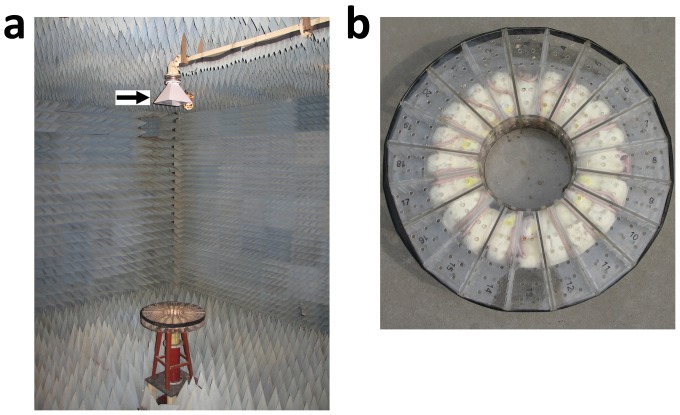
Microwave exposure system. (a) microwave radiation source.(b) the organic glass box in which rats were fixed to receive microwave radiation.

Animals in groups R, L, M, and H received microwave radiation. In brief, the microwave system was placed in a standard echoless dark chamber (length = 7/width = 6.5/height = 4), that has minimum reflected waves (Fig. 1a). Rats were fixed in an organic glass box (Fig. 1b), and the radiation table was rotated to assure whole body radiation. Rats were exposed to radiation with an average power density of 30 mW/cm^2^ for 15 min. In group C, animals were placed in the box but not exposed to radiation.

### Treatment with KFL

Starting on the day of radiation exposure, KFL was intragastrically administered once daily for 14 consecutive days. KFL was prepared by decoction ethanol extraction of the plants, yielding a clear paste of 1.4–1.5 fold concentration that was dried and crushed. The KFL was then dissolved in distilled water and mixed to prepare solutions of different concentrations. Rats in the low dose (L), medium dose (M), and high dose (H) groups were administered KFL intragastrically at 0.75, 1.5, and 3 g/kg/day, respectively. Rats in groups C and R were intragastrically treated with distilled water. Animals were weighed daily, and the dose of KFL was determined according to body weight.

### Electrophysiological examination

At 7 and 14 days (6 h after treatment discontinuation) after radiation, the animals were intraperitoneally anesthetized with 1% sodium pentobarbital at 30 mg/kg. The rats were then fixed in a supine position, and a multichannel physiologic recorder (Biopac MP-150; Goleta, CA, USA) and supporting analysis system were used for electrocardiography. The electrocardiogram (ECG) was used to determine the heart rate, J point shift (difference in voltage at J point and baseline), and changes in T wave amplitude.

### Histological and ultrastuctural examination of the heart

At 7 and 14 days (6 h after treatment discontinuation) after radiation, the animals were anesthetized, and the heart was harvested and fixed in 10% buffer formalin. After routine dehydration, embedding in paraffin, and sectioning, H&E staining was performed. Sections were observed under a light microscope (DM3000; Leica, Germany). Heart samples (1 mm^3^ cubes) of fresh tissues were fixed in 2.5% glutaraldehyde, sequentially processed with 1% osmium tetroxide, graded ethyl alcohols, and embedded in EPON618. Ultrathin sections cut onto copper mesh grids were stained with the heavy metals, uranyl acetate, and lead citrate for contrast. After drying, the grids were then viewed on a transmission electron microscope (TEM; HITACHI Ltd, Tokyo, Japan).

### Isolation of heart mitochondria

At 6 h after treatment discontinuation (7 days after radiation), mitochondria were extracted from heart tissue using mitochondrial extraction kit (Genmed Scientifics INC., Shanghai, China) according to manufacturer’s instructions. After a brief perfusion to rinse out the blood, tissues were minced and homogenized. The homogenate was centrifuged at 1500 g for 10 min, and the resulting supernatant was centrifuged at 10000 g for 10 min to pellet the mitochondria. The final mitochondrial pellet was resuspended in Reagent E (preservation solution). The mitochondria were fixedand observed by TEM.

### Detection of mPTP opening

At 6 h after treatment discontinuation (7 d after radiation), an mPTP fluorescence detection kit (Genmed Scientifics INC., Shanghai, China) was used to assess mPTP opening. Calcein was used to stain the mitochondria. This dye selectively aggregates inside the mitochondria, resulting in green fluorescence. The dye is released from the mitochondria when the mPTPs open. The change in mitochondrial fluorescence thus reflects the degree of mPTP opening. A laser scanning confocal microscope (LSM 510 META; ZEISS, Germany) was used to observe the sections, and fluorescence spectrophotometry was used to determine the fluorescence intensity of the mitochondria (Victor X; Perkin Elmer, Hamburg, Germany).

### Detection of myocyte mitochondrial membrane potential

At 6 h after treatment discontinuation (7 days after radiation), the mitochondrial membrane potential was measured using GENMED mitochondrial inner membrane fuction/membrane potential fluoremetry kit (Genmed Scientifics INC., Shanghai, China). Highly sensitive fluorescent carbocyanine dye was used to stain the mitochondria. The dye binds to the mitochondrial matrix, and the intensity of red fluorescence reflects the degree of mitochondrial electronegativity. A confocal laser scanning microscope (CLSM 510 META; ZEISS, Germany) was used to observe the sections, and a fluorescence spectrophotometer was used to detect the intensity of fluorescence (Victor X; Perkin Elmer, Hamburg, Germany).

### Immunohistochemistry

In brief, sections were incubated at 4°C overnight with an anti-connexin-43(Cx-43) primary antibody (1∶100, monoclonal antibody, Boster, China) or an anti-endothelial nitric oxide synthase (eNOS) primary antibody (1∶100, Bioworld, China), followed by incubation with biotinylated secondary antibodies and the streptavidin–peroxidase complex (ZSGB-Bio, China) at 1∶200 dilutions for 1 h at 37°C. Tissue sections were colorized with diaminobenzidine (ZSGB-Bio, China). Cx-43 and eNOS positive cells were analyzed with CMIAS imaging processing and analysis software (Beijing University of Aeronautics and Astronautics, China).

### Western blotting analysis

Protein concentrations of samples were determined using the BCA protein assay (Thermo Scientific, USA). After electroblotted onto polyvinylidene difluoride membranes (PVDF) (Millipore, USA), membranes were blotted by anti-voltage-dependent anion channel (VDAC) antibody at 1∶1000 dilution (Cell Signaling Technology, USA), or anti-GAPDH antibody (KangChen Biology, China), respectively. Images were scanned and analyzed by multiimage II system (Alpha Innotech, Silicon Valley, USA). The band signals of VDAC proteins were normalized to GAPDH.

### Statistical analysis

Data were expressed as mean ± standard deviation. SPSS version 16.0 was used for statistical comparisons of heart rate, J point shift, and T wave amplitudes. Comparisons between the radiation group and the control group were made using Student’s t-test; comparisons between groups L, M, and H and the radiation group were made using one-way analysis of variance.

## Results

### Electrophysiological cardiac changes after radiation and KFL treatment

As shown in Table 1, animals treated with radiation but no KFL (R group) demonstrated a significantly lower heart rate at 7 days after radiation treatment (P<0.01) compared to normal controls; the reduced heart rate remained at 14 days after radiation (P<0.05). In addition, the J point shift increased significantly in R group animals over that of normal controls at 7 days after treatment (P<0.05), but the T wave amplitude remained unchanged. When compared with the R group, animals in groups L (0.75 g/kg/day KFL), M (1.5 g/kg/day KFL), and H (3 g/kg/day KFL) demonstrated a significantly higher heart rate than normal controls at 7 days after radiation (P<0.01). A significant increase in heart rate was observed in groups M and H at 14 days after radiation (P<0.05). At 7 and 14 days after radiation, the J point shift increased to different extents, with the greatest increase observed in groups M and H (P<0.05). The amplitude of T waves remained unchanged. These observations suggest that microwave radiation can induce electrophysiological dysfunction of the heart, characterized by reduced heart rate and increased J point shift. KFL was observed to exert myocardial protective effects. Stronger protective effects were observed in groups M and L, but there was no significant difference between groups M and L. Thus, KFL has the optimal protective effort at the dose around 1.5 g/kg/day in this animal radiation model.

**Table 1:** The effects of KFL on the electrophysiological cardiac changes of the rats treated with HPM radiation.

Time point	Group	Index		
		Heart rate(times/min )( ×10^2^)	J point shift(mV)(×10^−2^)	T wave amplitude (mV) (×10^−2^)
7 days after treatment discontinuation(7 days after radiation)	C	4.06±0.18	1.24±0.68	6.73±1.09
	R	3.69±0.09**	1.81±0.40*	6.37±0.51
	L	3.89±0.08^ΔΔ^	1.49±0.48	6.53±0.66
	M	4.00±0.10^ΔΔ^	1.32±0.48^Δ^	6.63±0.82
	H	3.98±0.10^ΔΔ^	1.23±0.40^Δ^	6.77±0.67
6 h after treatment discontinuation(14 days after radiation)	C	3.99±0.22	1.17±0.40	6.54±0.52
	R	3.79±0.13*	1.64±0.47*	5.95±0.85
	L	3.84±0.22	1.35±0.41	6.12±0.72
	M	3.96±0.23^Δ^	1.19±0.41^Δ^	6.49±0.47
	H	3.95±0.20^Δ^	1.21±0.36^Δ^	6.43±0.64

Values are the mean ± SD. *P<0.05, **P<0.01, compared to normal control group; ^Δ^P<0.05, ^ΔΔ^P<0.01, compared to the radiation control group.

### Histological and ultrastructural changes in the heart

**Figure 2:**
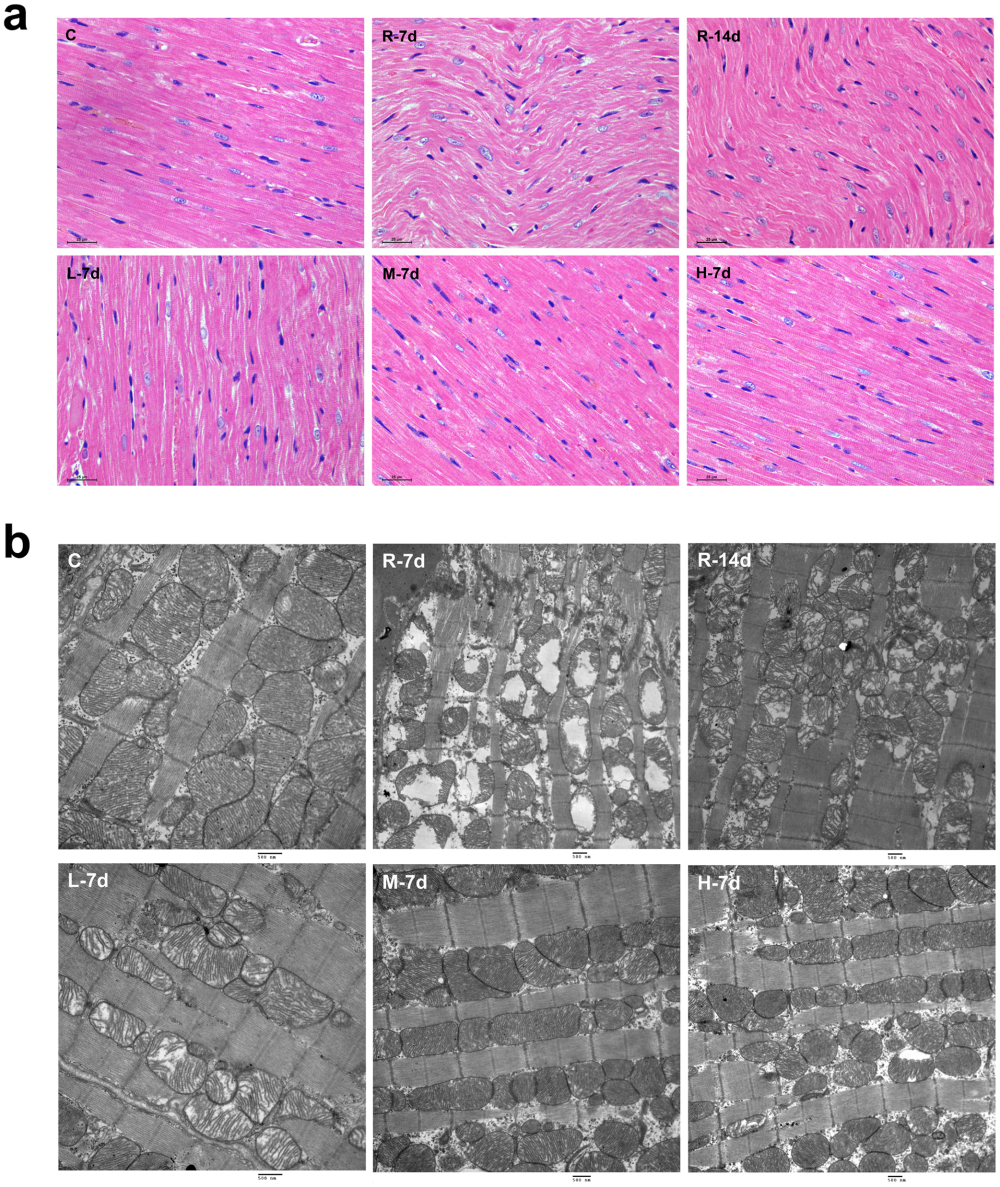
Histological and ultrastrucural changes in the heart (H&E staining, scale bar  = 25 µm; TEM, scale bar  = 500nm). (a) In the normal control group, myocytes were cylindrical, muscle fibers were in parallel arrangement and clear. At 7 days after radiation, the myocytes showed swelling, the muscle fibers were arranged in a wavy manner. At 14 days after radiation, myocytes showed mild swelling, the muscle fibers were arranged regularly. After 7 days of treatment with 0.75 g/kg/day KFL, myocytes showed mild swelling, the muscle fibers were arranged regularly. After treatment with KFL at 1.5g/kg/day and 3 g/kg/day, the myocytes were cylindrical, myocyte swelling was not observed, and the muscle fibers were regularly arranged. (b) In the normal control group, myofilaments were well-arranged and mitochondria had integrated structure. At 7 days after radiation, the arrangement of myofilaments were disordered and broken, and the mitochondria were swollen and vacuolized. At 14 days after radiation, the injuries of myocytes were slightly recovered. After treatment of irradiated animals with KFL at different concentrations, myocardial injury was recovered considerably, especially in the M group.

Our histological analysis of rat hearts showed that, in the normal control group, myocytes were cylindrical, muscle fibers were in parallel arrangement and clear, and the nuclei were oval and centrally located. At 7 days after radiation, myocytes were swelling, the muscle fibers were arranged in a wavy manner and were indistinct, and some nuclei showed pyknosis with dark staining. At 14 days after radiation, myocytes were mild swelling, the muscle fibers were arranged regularly, and no evidence of pyknosis was observed. After treatment of irradiated animals with KFL at different concentrations, myocardial injury was reduced to different extents. After 7 days of treatment with 0.75 g/kg/day KFL, myocytes showed mild swelling, the muscle fibers were arranged regularly, and the number of pyknotic nuclei was significantly lower than that in radiation controls. After treatment with KFL at 1.5 g/kg/day and 3 g/kg/day, the myocytes were cylindrical, myocyte swelling was not observed, the muscle fibers were regularly arranged and distinct, and the nuclei were oval and located at the center (Fig. 2a).

Pathological changes in the heart ultrastructure were observed by TEM. In the normal control group, myofilaments were well-arranged and mitochondria had integrated structure. At 7 days after radiation, the arrangement of myofilaments were disordered and broken, and the mitochondria were swollen and vacuolized. At 14 days after radiation, the injuries of myocytes were slightly recovered. After treatment of irradiated animals with KFL at different concentrations, myocardial injury was reduced, especially in the M group (Fig. 2b).

### Ultrastructural changes in isolated mitochondria

**Figure 3:**
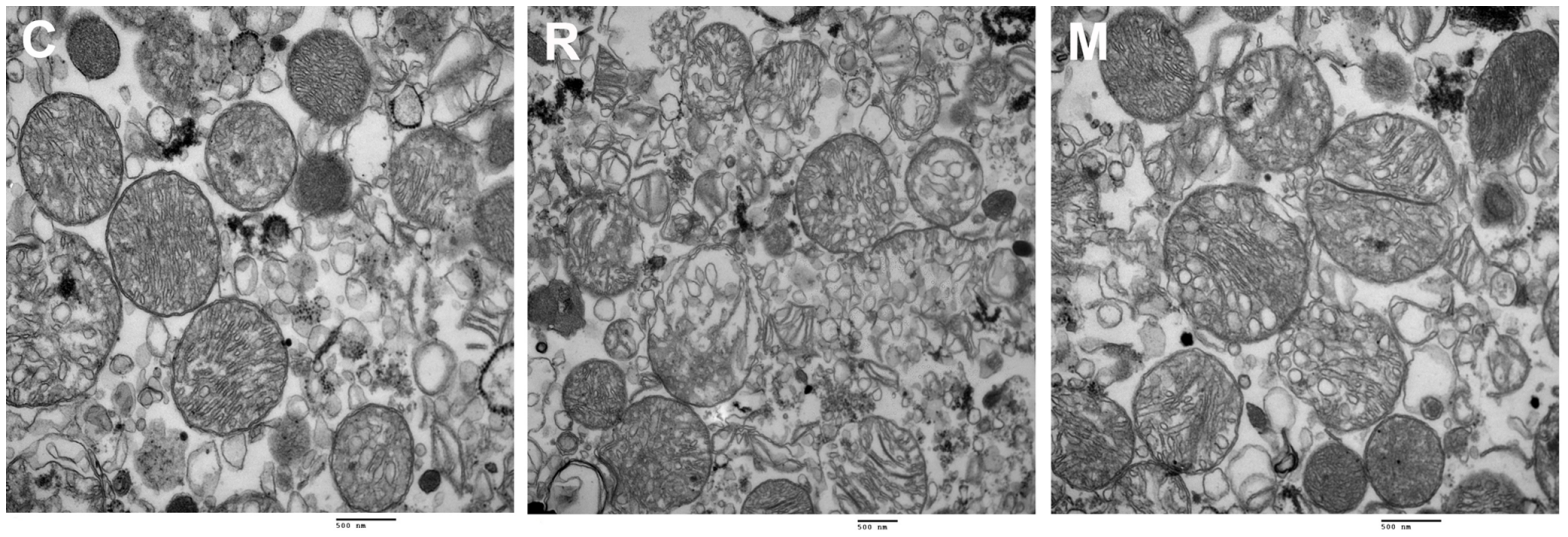
Ultrastructural changes in isolated mitochondria (TEM, scale bar  = 500 nm). In the normal control group, the mitochondria were of normal morphology, with an oval shape and intact structure. At 7 days after radiation, mitochondrial swelling and abnormal morphology (racket shaped) were observed, and cavitation, an absence of cristae, and rupture of the mitochondrial membrane were also noted. At 7 days after treatment of irradiated animals with KFL at 1.5 g/kg/day, the mitochondrial swelling was less and the mitochondria were oval and had normal structures.

We observed high numbers of mitochondria by electron microscopy analysis in C group and M group. In the normal control group, the mitochondria were of normal morphology, with an oval shape and intact structure. At 7 days after radiation, mitochondrial swelling and abnormal morphology (racket shaped) were observed, and cavitation, an absence of cristae, and rupture of the mitochondrial membrane were also noted. At 7 days after treatment of irradiated animals with KFL at 1.5 g/kg/day, the mitochondrial swelling was less visible and the mitochondria were oval and had normal structures (Fig. 3).

### mPTP opening and mitochondrial membrane potential in cardiac mitochondria

**Figure 4:**
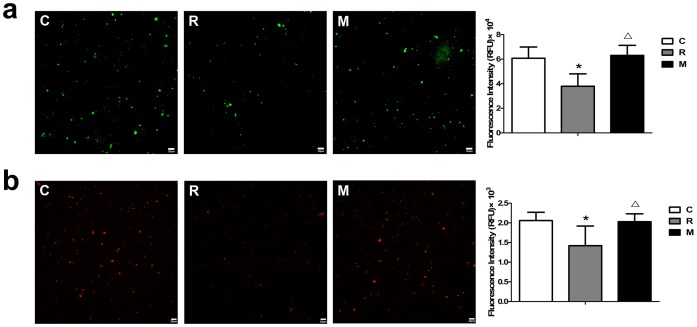
mPTP opening and mitochondrial membrane potential in cardiac mitochondria (fluorescence imaging with laser scanning confocal microscopy, scale bar = 20 µm). (a) Mitochondrial fluorescence had a high intensity in both normal control group and the radiation group after 7 days of treatment with 1.5 g/kg/day KFL. At 7 days after radiation, the fluorescence intensity was reduced in the radiation control group. (b) Fluorescence intensity was high in the mitochondria of both normal control group and the radiation group after 7 days of treatment with 1.5 g/kg/day KFL. At 7 days after radiation, the fluorescence intensity was reduced in the radiation control group. *P<0.05, compared with normal control group, ^Δ^P<0.05, compared with radiation control group.

Our laser scanning confocal microscopy experiments showed that control group has a high of red fluorescence reflecting the degree of mitochondrial electronegativity. At 7 days after radiation, the fluorescence intensity was significantly lower than that of the normal control group, suggesting the increased mPTP opening. After 7 days of treatment of irradiated animals with KFL at 1.5 g/kg/day, the fluorescence intensity was increased significantly compared to the radiation control group, suggesting a reduced mPTP opening. Fluorescence spectrophotometry confirmed that the fluorescence intensity at 7 days after radiation was significantly lower than that of the normal control group (P<0.05). After 7 days of treatment of irradiated animals with KFL at 1.5 g/kg/day, the fluorescence intensity was significantly higher than that of the radiation group (P<0.05), suggesting that treatment with KFL at 1.5 g/kg/day inhibited HPM-induced mPTP opening (Fig. 4a).

Next, we test mitochondrial membrane potential in cardiac mitochondria by laser scanning confocal microscopy analysis. The fluorescence intensity was high in the mitochondria of normal controls, suggesting a high mitochondrial membrane potential. At 7 days after radiation, the fluorescence intensity was reduced and the mitochondrial membrane potential was decreased. After 7 days of treatment of irradiated animals with KFL at 1.5 g/kg/day, the fluorescence intensity was significantly higher than that of the radiation control group, suggesting that the mitochondrial membrane potential was recovered. Fluorescence spectrophotometry analysis showed that the fluorescence intensity was significantly lower at 7 days after radiation than that of normal controls (P<0.05). After 7 days of treatment of irradiated animals with 1.5 g/kg/day KFL, the fluorescence intensity was significantly higher than that of the radiation control group (P<0.05), suggesting that 1.5 g/kg/day KFL reverted the HPM-induced reduction in mitochondrial membrane potential (Fig. 4b).

### Cx-43 and eNOS expression in the heart

**Figure 5:**
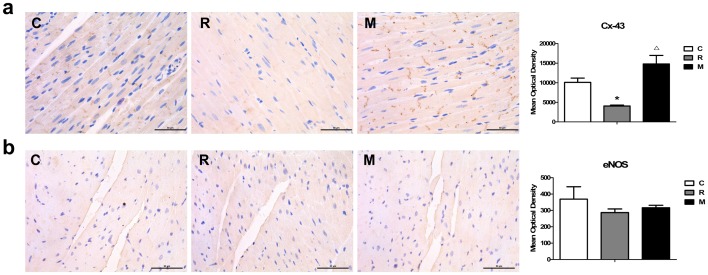
Effects of KFL on myocardial Cx-43 and eNOS expression in the heart induced by microwave radiation (Immunohistochemistry, scale bar  = 50 µm). Shown are myocardial immunohistochemistry analyses of (a) Cx43, (b) eNos. Cx43 was decreased, and eNos was increased in response to microwave radiation, and all of these changes were abrogated by KFL. *P<0.05, compared with normal control group, ^Δ^P<0.05, compared with radiation control group.

Cx-43 is a major structural protein found in the gap junctions of the ventricular myocardium and a major determinant of its electrical properties. To further investigate the electrophysiological changes in the heart after KFL treatments, the level of the Cx-43 protein was evaluated by immunohistochemistry. The Cx-43 expression was found to be lower in the microwave cardiomyocytes compared to that in normal group (P<0.05). KFL markedly attenuated the microwave-induced downregulation of total Cx-43 protein expression at 7 day (P<0.05) (Fig. 5a).

To further explore the possible role of eNOS during KFL treatment, we investigated myocardial eNOS expression by immunohistochemistry analysis. However, there were no significant changes of eNOS expression in myocardial cell and endothelial cell after microwave radiation or KFL administration (Fig. 5b).

### Protein immunoassay by Western blotting

**Figure 6:**
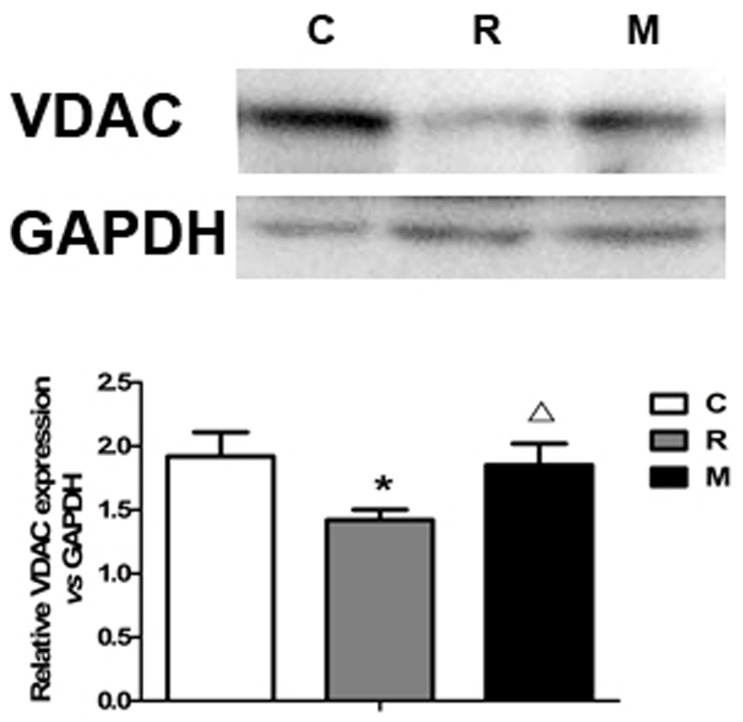
The effect of KFL on mitochondrial protein levels of VDAC in the rats treated with radiation. Representative Western blots of VDAC are shown for each group. *P<0.05, compared with normal control group; ^Δ^P<0.05, compared with the radiation control group.

Next, western blotting analysis was performed in order to semiquantify mPTP proteins such as VDAC. Microwave exposure induced a significant decrease of VDAC (P<0.05). KFL with 1.5 g/kg/day was able to restore the protein levels to control values at 7 days after exposure (P<0.05) (Fig. 6).

## Discussion

The HPM provokes acute myocardial damages of structure and function in lab animals. It has been shown that, when rats were treated with HPM radiation at 100 mW/cm^2^ for 6 minutes, disappearance and rupture of filaments, together with mitochondrial swelling and cavitation could been observed at 1 and 7 days after radiation [16]. For macaques, with radiation at 11 mW/cm^2^, their heart rates were reduced immediately after radiation, and the amplitudes of R and T waves were reduced significantly at 1 day after radiation [17]. In the present study, we observed reduced heart rates, a J point shift, swelling and wave-like arrangement of myocytes, and mitochondrial swelling and rupture in rats at 7 days after HPM radiation. These observations indicate that changes in myocardial electrophysiology and structure are associated with myocardial injury, although the specific pathways involved in the pathogenesis of microwave-radiation–induced myocardial injury are unknown.

Mitochondria are extremely important organelles in myocytes, accounting for 40–60% of the cell volume [18]. The mPTP multi-protein complex in the mitochondrial membrane is a key component in mitochondrial signal transduction [19]. Excessive mPTP opening leads to a reduction in the mitochondrial membrane potential and is associated with numerous heart diseases [20], [21]. Under normal physiological condition, mPTPs are closed. The inner mitochondrial membrane is selectively permeable, allowing passage of some metabolic substrates and ions but not others. Under certain conditions, including calcium overload, oxidative stress, and impaired signal transduction, the mPTPs open and thus allow larger molecules to enter the mitochondria, causing increased osmolality of the mitochondrial matrix. This condition may lead to mitochondrial swelling, rupture of the outer mitochondrial membrane, leakage of pro-apoptotic proteins (such as cytochrome C) into the cytoplasm, abolition of the mitochondrial transmembrane potential, uncoupling of the respiratory chain from oxidative phosphorylation, discontinuation of Adenosinetriphosphate (ATP) synthesis, finally resulting in cell death [22], [23].

In recent years, mPTP opening has been considered a sensitive therapeutic target for testing cardio protection during numerous cardiac pathological conditions. A study from Sun et al. [13] showed that early ischemia/reperfusion injury was caused by mPTP opening secondary to calcium overload, and post-ischemic treatment prevented calcium overloading and closed the mPTPs, exerting cardioprotection. Piot et al. [14] found that patients with ST segment elevation myocardial infarction who were treated with an mPTP inhibitor (cyclosporin A) had significantly reduced CK levels and myocardial infarct ratios, compared to those without treatment. In the present study, our results showed that at 7 days after microwave radiation of rats, the fluorescence intensity of stained mitochondria was reduced, suggesting opening of mPTPs and a consequent reduction in mitochondrial membrane potential. Thus, a potential effective treatment for microwave radiation could revert mPTP openings induced by microwave radiation as well.

Indeed, we now find that “ KFL”, a compound from traditional Chinese medicine protected rats from HPM-induced myocardial injury and also reverted mPTP opening induced by microwave radiation. After treatment of irradiated rats with 1.5g/kg/day KFL for 7 d, the heart rate recovered significantly (P<0.01), the J point shift was also reverted significantly (P<0.05), and the structures of myocytes and mitochondria were of normal morphology. Moreover, the fluorescence intensity of stained mitochondria was increased by KFL treatment, suggesting inhibition of mPTP opening and preservation of the mitochondrial membrane potential.

We further investigated the molecular mechanism of KFL protection. First, in our study, the microwave-induced decrease in Cx-43 protein expression was significantly reversed by KFL, which indicated Cx-43 dysfunction in cardiomyocytes may contribute to the pathogenesis of electrophysiological changes in the heart after treatments. Second, we also found that microwave radiation had not affect eNOS protein expression in the heart, which was not changed after KFL administration, neither. These results indicated that microwave may not cause cardiac damage and vascular dysfunction through eNOS expression, while KFL may not ameliorate cardiac and vascular remodeling through eNOS expression. Finally, because the mPTP is composed of VDAC in the outer membrane, we observed the decreased expression of mitochondrial VDAC after microwave radiation, and it was reverted by KFL with 1.5g/kg/day. Thus, the protective effects of KFL on mitochondria, particularly on mPTP, may be mediated by modulation of pore proteins. These findings support that mPTP plays an important role in the protective effects of KFL against microwave-radiation–induced myocardial injury. However, whether mPTP is the final effecter, how KFL acts on mPTP, and what other signaling pathways are involved in this action remain to be determined by further studies.

Traditional Chinese medicines have been widely used for the treatment of cardiovascular diseases, with many favorable results [8], [11]. However, determining optimum treatment dose of traditional Chinese medicine compound is still a challenge partially because of the nature of these compounds. In this study, we explored the optimal dose of KFL’s myocardio protective effects in our animal model. The efficacy of KFL was highest in groups M (1.5 g/kg/day) and H (3.0 g/kg/day). However, the difference in the therapeutic efficacy between these two groups was not significant. Thus, the lower dose of KFL is enough to execute its protective effects in rat with microwave-radiation–induced myocardial injury. We proposed that a dose of 1.5 g/kg/day KFL is optimal for the protection against microwave-radiation–induced myocardial injury in rats. The optimal biological dose of KFL was also examined here for the benefit of potential preclinical trials.
